# Investigating the Impact of *Nosema* Infection in Beehives on Honey Quality Using Fluorescence Spectroscopy and Chemometrics

**DOI:** 10.3390/foods14040598

**Published:** 2025-02-11

**Authors:** Mira Stanković, Miloš Prokopijević, Filip Andrić, Tomislav B. Tosti, Jevrosima Stevanović, Zoran Stanimirović, Ksenija Radotić

**Affiliations:** 1 Institute for Multidisciplinary Research, University of Belgrade, 11030 Belgrade, Serbia; mira.mutavdzic@imsi.rs (M.S.); milos.prokopijevic@imsi.rs (M.P.); 2 Faculty of Chemistry, University of Belgrade, 11158 Belgrade, Serbia; andric@chem.bg.ac.rs; 3 National Institute of the Republic of Serbia, University of Belgrade Institute of Chemistry, Technology and Metallurgy, Studentski Trg 12-16, 11158 Belgrade, Serbia; tosti@chem.bg.ac.rs; 4 Faculty of Veterinary Medicine, University of Belgrade, 11000 Belgrade, Serbia; rocky@vet.bg.ac.rs (J.S.); zoran@vet.bg.ac.rs (Z.S.)

**Keywords:** honey, *Apis mellifera*, *Nosema ceranae*, physico-chemical properties, sugar composition, fluorescence spectroscopy, chemometrics

## Abstract

This study investigates the impact of *Nosema* infection in beehives on the physico-chemical and biochemical properties and spectral characteristics of honey as indicators of honey quality. Comprehensive analyses were performed on honey samples from hives with varying levels of *Nosema* infection, examining water content, free acidity, optical rotation, electrical conductivity, sugar composition, catalase activity, and pollen content. Honey from highly infected hives showed higher water content (up to 17.3%), lower optical rotation, reduced electrical conductivity, decreased glucose levels, and increased sucrose levels. Principal component analysis (PCA) identified distinct clustering of samples based on infection levels, with changes in the sugar profile, particularly higher phenolic compounds, correlating with increased infection levels. Fluorescence spectroscopy combined with PARAFAC modeling identified proteins and phenolic compounds as key discriminators of honey from infected hives. Correlation and PLS modeling further demonstrated strong relationships between spectral features and honey properties, including catalase activity and pollen content. This research presents a novel approach to evaluating the impact of Nosema infection on honey quality by integrating physico-chemical and biochemical analyses and sugar composition profiling with advanced spectroscopic techniques. These insights are invaluable for improving bee health monitoring practices and advancing sustainability in the beekeeping and honey production industries.

## 1. Introduction

Honey, a remarkable product of honeybee activity, has long been appreciated for its nutritional, medicinal, and economic value. Its composition, which includes a variety of sugars, enzymes, antioxidants, and minerals, is largely influenced by factors such as the floral source, environmental conditions, and the health of the bee colony. Among the key determinants of honey quality are its physico-chemical properties, such as color, pH, viscosity, free acidity, specific optical rotation, and the concentration of sugars like fructose, glucose, and sucrose [1]. These properties not only define the sensory and nutritional qualities of honey but also provide insights into the quality and authenticity of the product.

Recent concerns over the alarming decline in honeybee populations, exacerbated by colony collapse disorder (CCD), have brought global attention to the broader environmental and ecological impacts of bee health. A significant factor contributing to the weakening of bee colonies is the infection of bees by the microsporidian parasite *Nosema ceranae*. This pathogen has been associated with a decline in colony productivity, survival rates, and overall colony health, influencing honey production and potentially altering its composition [2]. The presence of *Nosema* infection in beehives could thus lead to changes in honey’s physico-chemical and biochemical properties, offering a potential link between bee health and honey quality [3].

In this context, fluorescence spectroscopy has emerged as a powerful analytical tool to study the chemical composition and quality of honey [4]. This technique allows for the identification of specific molecular signatures in honey, including those related to sugars and other bioactive compounds, and can provide insights into the impact of factors such as disease and environmental stress on honey composition. To further analyze these complex relationships, advanced statistical and chemometric techniques like Parallel Factor Analysis (PARAFAC) and Partial Least Squares (PLS) regression are being increasingly applied [5,6,7]. These methods allow for the multivariate analysis of honey’s physico-chemical properties, providing detailed correlations between different chemical components, environmental factors, and potential health threats to bee colonies.

This research aims to investigate the physico-chemical and biochemical properties and sugar composition of honey from beehives infected with *Nosema*. Using fluorescence spectroscopy, PARAFAC, PLS, and correlation analysis, we seek to explore the relationship between honey composition and colony health, shedding light on the potential markers of bee health and the impact of *Nosema* infection on honey quality [8]. By gaining a deeper understanding of these interactions, this study contributes to broader efforts to address colony collapse disorder and improve honey production sustainability [9]. The aim was to evaluate whether spectral analysis combined with chemometrics could serve as a rapid and sensitive method for assessing honey quality from infected hives. This approach addresses the limitations of traditional standard methods, which are often time-consuming and require extensive sample pre-processing.

This research presents a novel approach to evaluating the impact of Nosema infection on honey quality by integrating physico-chemical and biochemical analyses and sugar composition profiling with advanced spectroscopic techniques. Unlike traditional methods that are time-intensive and require extensive sample pre-processing, this study leverages fluorescence spectroscopy, PARAFAC, PLS, and correlation analysis to identify potential markers of bee health and Nosema-induced changes in honey composition. By uncovering specific interactions between Nosema infection, honey composition, and spectral characteristics, this research contributes valuable insights into mitigating the effects of colony collapse disorder and enhancing the sustainability of honey production.

## 2. Materials and Methods

### 2.1. Samples

In 2018, eight honey samples were collected from the apiary at the Faculty of Veterinary Medicine in Belgrade, Serbia. Along with the honey samples, bees were also collected for Nosema infection assessment. Microscopic examination was used to detect Nosema ceranae spores, and the infection levels were quantified. Pollen analysis indicated that the honey samples were multifloral.

### 2.2. Microscopic Detection of N. ceranae Spores and Quantification of the Degree of Infection

A microscopic examination of bee samples was carried out to detect Nosema spores following the guidelines of the World Organization for Animal Health [10]. Thirty adult foraging bees were collected directly from the entrance of each hive and then preserved by freezing for subsequent analysis. Each bee was then macerated in 2–3 mL of water, and the resulting suspension was analyzed under a microscope at 400× magnification.

### 2.3. Physico-Chemical Characteristics of the Honey

The physico-chemical parameters generally analyzed to assess the quality and authenticity of honey include electrical conductivity, moisture content, specific optical rotation, free and total acidity, and pH value [11].

#### 2.3.1. Determination of Electrical Conductivity

The electrical conductivity of the honey solution in distilled water was determined using the method recommended by the International Honey Commission (IHC) and is defined as the conductivity of a 20% aqueous solution of honey’s dry substance at 20 °C. Electrical conductivity is measured using a conductivity cell by measuring the electrical resistance, and the result is expressed in millisiemens per centimeter (mS/cm) [11].

A 20.0 g equivalent of anhydrous honey was dissolved in distilled water, and the solution was quantitatively transferred into a 100 mL volumetric flask, which was then filled to the mark with distilled water. An aliquot of 40 mL of this solution was placed in a beaker and thermostated at 20 °C, and the electrical conductivity was measured using a conductometer (Jenway Conductivity Meter 4310, St Neots, UK), which was calibrated with a standard solution (Reagecon (Shannon, Ireland) 1413 ± 1% μScm^−1^ at 25 °C). The conductivity is expressed in mScm^−1^.

#### 2.3.2. Determination of Water Content

The water content in the honey was determined according to the method of the International Honey Commission [11]. It was determined by the refractive index using an Abbe refractometer (Atago^®^ 1T Abbe refractometer, Tokyo, Japan) in comparison with a reference standard (Refractive index standard kit 2 (Toluol nD20 = 1.4969/Water nD20 = 1.3330) Merck KGaA, Frankfurt, Germany) at 20 °C. The obtained refractive index values were correlated with the values for water from the Chataway table to determine the final water content.

#### 2.3.3. Free Acidity of Honey

Free acidity was measured using the method outlined by the International Honey Commission (IHC). The honey sample (10 g) was dissolved in 75 mL of distilled water, and the resulting solution was titrated with a 0.1M NaOH standard solution to a pH of 8.6, using a pH meter (WTW 154 Inolab).

#### 2.3.4. Determination of Specific Optical Rotation

Carbohydrates are optically active substances that rotate the plane of polarized light. This property is the basis for the method of measuring the specific optical rotation of carbohydrates in honey. The specific angle of rotation is obtained by measuring the rotation angle of the analyzed aqueous honey solution [11], and the total optical rotation depends on the concentrations of the sugars present. The optical rotation of fructose is negative, while glucose has a positive rotation.

Specific optical rotation (SR), [*α*]20 °C, is the angle of rotation of polarized light measured at the sodium D-line wavelength (λ = 589.3 nm) at 20 °C in a 1 dm cell containing 1 g/mL of the substance.

A sample of 12 g of honey (equivalent to 10 g of dry matter) was dissolved in distilled water and transferred to a 100 mL volumetric flask. Then, 10 mL of Carrez I reagent was added, followed by Carrez II reagent [11]; the solution was made up to the mark and left for 24 h. After that, the solution was filtered and thermostated at 20 °C. A 2 dm polarimeter tube was filled with the thermostated solution, and the rotation angle (α) was read on the polarimeter.

### 2.4. Determination of Sugars in the Honey

For the identification and quantification of sugars in honey, high-efficiency ion chromatography with pulsed amperometric detection (HPAEC/PAD) from Dionex (model ICS 3000) was used. A CarboPac PA100 analytical column, a hydroxide-selective anion-exchange column (4 × 250 mm, made of polyetheretherketone, PEEK), was used to separate the sugars. The stationary phase consists of 9 μm diameter granules made of copolymers of ethylvinylbenzene and divinylbenzene (55% by mass), while the ion-exchange layer is derivatized with alkanol–quaternary ammonium groups. A pre-column (CarboPac PA100 Guard column 4 × 50 mm) was used to further remove impurities from the honey sample. A flow rate of 0.7 mL/min was set, and three mobile phases were used for elution (600 mM NaOH (A), 500 mM CH3COONa (B), and ultra-pure H2O (C)) with a gradient program. A pulsed amperometric (electrochemical) detector was used for sugar analysis in honey samples: an electrochemical cell with an adjustable potential, a Ag/AgCl reference electrode, and a Au working electrode. Instrument control, data acquisition, and analyses were performed using the Chromeleon software package (version 6.80) (Thermo Fisher Scientific, Dionex, Sunnyvale, CA, USA).

For each sample, about 0.2 g of homogenized honey was dissolved in ultra-pure water at a concentration of 0.25 g/L. The resulting solution was then passed through a syringe filter (13 mm, PTFE membrane, 0.45 μm) and transferred to vials for the autosampler. The sugar content was expressed in g/kg as the average value of three measurements.

### 2.5. Biochemical Analysis

#### 2.5.1. Determination of Catalase Activity in the Honey Samples

Catalase activity in the honey samples was analyzed using the method described by Huidobro et al. [12]. Triplicate honey samples were dissolved in 0.015 M phosphate buffer (pH 7) and dialyzed against the same buffer for 22 h at 4 °C. Activity was determined by measuring the reduction rate of hydrogen peroxide (H_2_O_2_) spectrophotometrically with a Shimadzu UV-160 spectrophotometer (Kyoto, Japan). The reaction mixture consisted of o-dianisidine, peroxidase, and phosphate buffer (pH 6.1), to which a 200 µL aliquot of the dialyzed honey sample was added. The reaction was terminated by adding HCl, and absorbance was measured at 400 nm using 1 cm cuvettes at room temperature. Catalase activity was expressed as U/mg of protein, with results representing the average of three independent measurements per sample.

#### 2.5.2. Determination of Diastase Activity in the Honey Samples

Diastase activity in the honey was determined using a GBC UV-Visible Cintra 6 Spectrometer (Dandenong, Australia, Part Number: 01-0940-00), with absorbance measured at 660 nm. The analysis followed the Schade method [13], in accordance with the recommendations of the International Honey Commission [14]. Results were expressed as the diastase number (DN) in Schade units.

#### 2.5.3. Determination of Total Phenolic Content (TotPhC)

The honey samples were prepared following an adapted version of Gašić’s method [15]. A 5 g honey sample was dissolved in 10 mL of distilled water at room temperature, transferred to a 50 mL volumetric flask, and diluted to volume with ultrapure water. Total phenolic content was assessed spectrophotometrically using the modified Folin–Ciocalteu method [16]. Briefly, 0.3 mL of the honey solution was mixed with 6 mL of deionized water and 0.5 mL of Folin–Ciocalteu reagent and then incubated for 6 min at room temperature. Afterward, 3 mL of a 20% sodium carbonate solution was added, and the mixture was heated at 40 °C for 30 min. The absorbance was recorded with the aid of a Shimadzu UV-160 spectrophotometer (Kyoto, Japan). A calibration curve was prepared using gallic acid as the standard within the range of 0 to 250 mg/L. The blank solution consisted of deionized water and Folin–Ciocalteu reagent. The results were expressed as gallic acid equivalents (GAE) per kilogram of honey.

#### 2.5.4. Determination of Total Protein Content (TotPrC)

The total protein content was assessed using the Bradford method [17]. The honey samples (5 g) were diluted with 10 mL of distilled water, and 5 µL of the solution was mixed with 200 µL of Coomassie Brilliant Blue, forming a protein–dye complex. After 5 min incubation, absorbance was measured at 595 nm. A standard curve was created using bovine serum albumin (10–100 µg/0.1 mL) for calibration. The total protein content was subsequently determined and reported as grams per kilogram of honey.

### 2.6. Fluorescence Spectroscopy and Chemometrics

Fluorescence spectra of the honey samples were recorded using an Fl3-221 P spectrofluorometer (Jobin Yvon, Horiba, Palaiseau, France), featuring a 450 W Xe lamp and a photomultiplier tube for enhanced detection sensitivity. The sample was placed in a solid sample holder in the front-face configuration. To reduce light reflections, scattered radiation, and depolarization effects, the incident light angle was set at 22.5°. Rayleigh masking was applied to minimize the impact of Rayleigh scattering from the solid sample, enhancing measurement sensitivity and accuracy [18,19]. Fluorescence emission spectra were recorded between 280 and 550 nm, with excitation wavelengths from 270 to 370 nm, producing the excitation–emission matrix (EEM) for subsequent statistical analysis. The integration time was set to 0.1 s, with excitation and emission increments of 5 nm and 1 nm, respectively. Both excitation and emission slits were configured with a spectral bandwidth of 2 nm.

#### 2.6.1. Data Analysis and Modeling of Spectral Features

Fluorescence data for each honey sample were collected as excitation-emission matrices (EEMs). Parallel factor analysis (PARAFAC) and partial least squares regression (PLS) were conducted using the PLS Toolbox v. 7.0.3 (Eigenvector Research Inc., Manson, WA, USA). Prior to PARAFAC analysis, the EEMs were preprocessed to eliminate Rayleigh scattering and other spectral artifacts. Additionally, empty cells were filled with missing data (non-assigned values) rather than zeros to prevent distortion of the PARAFAC solutions.

#### 2.6.2. PARAFAC

The excitation-emission matrices (EEMs) of the honey samples were organized into three-way data arrays for PARAFAC analysis. A detailed description of PARAFAC can be found in Bro [20], but a brief explanation is provided herein. PARAFAC decomposes a three-way data set into trilinear components, with the number of components corresponding to the number of fluorophores present in the samples. Decomposition is carried out using an algorithm that minimizes the sum of squared residuals through a least squares approach (Equation (1)):

(1) $$x_{ijk}=\sum_{h=1}^{m}a_{ih}b_{jh}c_{kh}+e_{ijk}$$

In this equation, *x_ijk_* represents an element of the three-way EEM array, which corresponds to the measured fluorescence of sample *i* at emission wavelength *j* and excitation wavelength *k*. Each element is decomposed into a score (*a_ih_*) and the corresponding emission (*b_jh_*) and excitation (*c_kh_*) loadings for each of the h PARAFAC components, which represent the fluorophores in the sample. The residuals (*e_ijk_*) reflect the values not accounted for by the model, based on the selected number of components.

Since the number of components is not predetermined, the PARAFAC model must be validated. The model’s validity is ensured by minimizing the sum of squared residuals, leading to a unique solution. To confirm the model, split-half was employed [21,22]. If the correct model has been built from the data, splitting the data in half should independently yield two PARAFAC solutions with the same number of components and similar or identical emission and excitation properties as the original model. Model quality was also assessed through parameters like core consistency and the percentage of explained variance. For a fully developed PARAFAC model, core consistency should be 100%. The optimal number of components is determined by identifying a sudden drop in core consistency as the number of components increases.

#### 2.6.3. Correlation Analysis

Correlation analysis was conducted using the basic Data Analysis add-in for Microsoft Excel 365, where Pearson’s correlation coefficient was calculated between the PARAFAC scores and the other variables under study. The statistical significance of these correlations was determined using Student’s *t*-test.

#### 2.6.4. PLS

PLS is a linear approach to the modeling of dependent variables (here the PARAFAC scores) and a series of independent variables (in this case various physico-chemical properties). The method transforms the original variables into a series of latent variables (PLS components) that are most suitable for predicting the dependent one. In this way, a high-dimensional problem is reduced, which is of particular importance when the variables significantly outnumber the samples. PLS was performed through the implementation of the SIMPLS algorithm. Prior to analysis, the data were standardized by mean-centering and scaling to unit standard deviation. Due to the small number of honey samples, a double cross-validation (CV) approach was employed to select the optimal number of PLS components and estimate the predictive performance of the PLS models, following the methodology of Varmuza and Filzmoser [23]. The entire dataset was divided into five independent training and test sets in the outer CV loop using Venetian blinds (VB) resampling. Each training set was further split into calibration and validation subsets using leave-one-out resampling in the inner CV loop. This ensured that each honey sample was used in the calibration, validation, and test sets, but never simultaneously. The optimal model complexity was determined by selecting the number of PLS components that minimized the root mean square cross-validation error (RMSECV). Performance parameters calculated from the outer loop were then used to assess prediction ability, with RMSEPRED and R^2^PRED as the key metrics. In order to improve model performance, variables with variable importance to projection (VIP) scores lower than 1 were excluded in a repeated stepwise procedure. After each step, model performance parameters were monitored, and if no improvement was noticed, the procedure was stopped. Also, honey samples that exhibited an outlying effect and could significantly contribute to model deterioration were initially removed. The regression coefficients and VIP scores of the final models were further used for the estimation of individual contributions of physico-chemical properties to the modeling of fluorescence spectra characteristics.

## 3. Results

The results of the physico-chemical parameters of honey samples originating from hives infected with *Nosema* are shown in Table 1.

The results of the sugar content in the honey samples originating from hives infected with *Nosema* are shown in Table 2.

## 4. Discussion

### 4.1. Physico-Chemical Characteristics

Although the obtained results indicate that the samples mostly meet the prescribed honey quality requirements [24], it is obvious that there is a trend of changing physico-chemical parameters with increasing *Nosema* infection levels (Table 1). The results show that with increasing *Nosema* infection levels, the water content in the honey samples also increases (Table 1). The lowest water content (13.10%) was measured in honey from a hive where the bees were not infected with *Nosema* spores, while the highest water content (17.30%) was found in honey from a hive where all bees were infected. It is known that water content depends on the botanical origin of the nectar, climatic conditions during the season, the degree of maturity of the honey in the hive, beekeeping practices, and honey storage conditions [25,26]. All honey samples showed appropriate maturity levels, in accordance with international water content standards, which should be below 20% [27].

The free acidity of honey decreased with the increase in *Nosema* infection levels in the corresponding hives (Table 1). The highest free acidity value (31.83 meq/kg) was found in honey from a hive with uninfected bees, while lower values were observed in highly infected hives. Free acidity is closely related to the spoilage process of honey. Increased free acidity values (above 50 meq/kg) in honey samples are an indicator of sugar fermentation into acids [28], which was not the case in our samples.

When observing the absolute values of the specific optical rotation of honey, it can be noted that they decrease as the infection level of the hives increases. The optical rotation of honey depends on the sugar composition and concentration, as well as whether the bees produced the honey from honeydew (which rotates polarized light to the right—right-handed) or nectar (which rotates polarized light to the left—left-handed). The optical rotation values of our samples are mostly negative, indicating that the honey originates from nectar [29,30].

The electrical conductivity of the honey samples decreases with the increase in the infection levels of the hives. The sample from the hive where bees were not infected had the highest electrical conductivity value (0.600 mS/cm), while honey from highly infected hives had significantly lower electrical conductivity (0.230 mS/cm). Electrical conductivity is a parameter frequently used in routine honey quality control and can be considered a valid criterion for determining the botanical origin of honey samples and distinguishing honey from nectar and honeydew. It depends on minerals, organic acids, and protein content in honey [31].

### 4.2. Sugars in the Honey

Highly efficient ion chromatography with pulsed amperometric detection (HPAEC/PAD) was applied to determine the sugar profile, as shown in Table 2. Fructose and glucose were identified as the most prevalent sugars in all tested samples. The content of both sugars is within the limits set by EU legislation [27]. Increased glucose levels (343.60–311.90 g/kg) were observed in honey samples from hives with lower *Nosema* infection levels (0–30%). For honey samples from hives with higher infection (43.33–100%), glucose values were lower (246.50–278.30 g/kg). The sucrose content was twice as high in a sample from a hive where all bees were infected with *Nosema* spores compared to a sample from an uninfected hive. However, the other sugar content results showed variability between hives with different infection levels.

**Figure 1:**
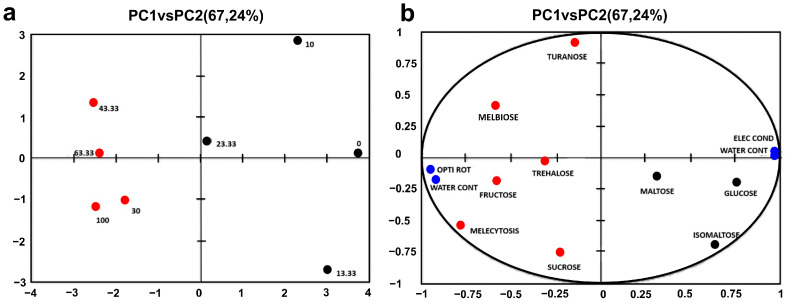
Principal component analysis (PCA) of honey samples based on physico-chemical parameters from hives with varying levels of *Nosema* infection: (**a**) score plot and (**b**) loading plot.

Figure 1 shows the results of the principal component analysis (PCA), including the score plot and loading plot. The PCA applied to the physico-chemical analysis results and sugar content of the honey samples resulted in a model explaining 67.24% of the total variation, with 48.13% attributed to PC1 and 19.10% attributed to PC2. Information from the remaining variables was not significant for the analysis. Samples marked in red on the score plot represent honey from highly infected hives and are located on the left side of the PCA score plot. Along the PC1 axis, honey from highly infected hives, characterized by lower absolute values of optical rotation and higher water content, is separated. The content of total sugars, such as fructose, melbiose, and melezitose, is higher in honey from more infected hives compared to samples from less infected hives. On the other hand, glucose and isomaltose are more abundant in honey from less infected hives. The right side of the score plot corresponds to samples (marked in black) from less infected hives. The main contributing factors in this case are electrical conductivity and free acidity, which are higher in honey samples from less infected hives, as well as glucose and isomaltose, which are also present in higher amounts in these samples compared to those from more infected hives.

These results are in accordance with those obtained from the DSC analysis, which indicated changes in the sugar environment, the main component in honey samples [32]. This can be linked to the increased water content in samples from hives with higher infection levels (Table 1). The physical properties of honey, such as crystallization and viscosity, depend on the water content in the honey [33,34]. During crystallization, sugars convert into crystalline form, which increases the water content in the liquid phase of the honey [35].

### 4.3. PARAFAC Results

**Figure 2:**
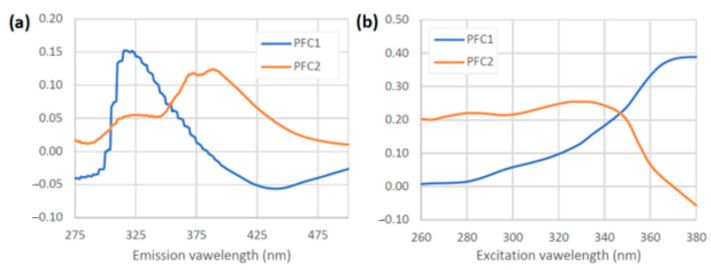
Emission (**a**) and excitation (**b**) loading vectors for the two components (PFC1 and PFC2) in the PARAFAC model.

The PARAFAC models were constructed by gradually increasing the number of components, ranging from 1 to 5. Examination of the residuals and changes in core consistency indicated that the spectral data were best described by two components. The loading vectors for these two components, obtained through EEM decomposition, are shown in Figure 2. These components are believed to represent the inherent pure emission (a) and excitation (b) spectra of the characteristic honey fluorophores.

For the first PARAFAC component (PFC1), the emission loading vector exhibits a maximum at 325 nm, while the corresponding excitation loading vector peaks above 360 nm. Based on previous studies [7,36], this component is attributed to the protein fluorophore in honey.

The second component (PFC2) displays a minor emission peak at 325 nm, accompanied by two distinct maxima in the range of 370 to 400 nm. The excitation loading vector of PFC2 displays significant intensity between 260 and 345 nm. This is likely associated with the phenolic compounds found in honey samples [7,36,37,38,39].

**Figure 3:**
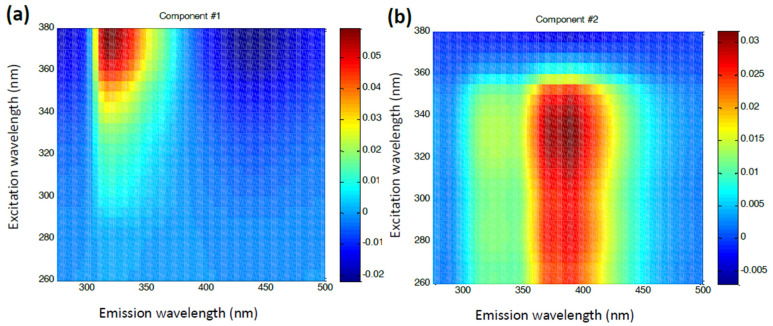
Heatmaps of the EEM loading vectors for the first (**a**) and second (**b**) PARAFAC component corresponding to the proteins and phenolic compounds in the honey samples, respectively.

The emission and excitation characteristics of the loading vectors for the first and second components are further illustrated in Figure 3a,b as EEM heatmaps. These heatmaps clearly highlight regions corresponding to protein fluorophores (upper left corner of the map, 300–350 × 340–380 nm) and phenolic fluorophores (middle region of the map, 350–425 × 260–360 nm).

### 4.4. Correlation Analysis

To investigate potential correlations between spectral features and the physico-chemical properties of the honey samples, particularly the infection degree (%), a correlation matrix was generated using Pearson’s correlation coefficient. This matrix was calculated for the PARAFAC scores of both components, their ratios (PFSC1/PFSC2 and PFSC2/PFSC1), and the other properties under study. The results are presented in the correlation table (Supplementary Data Sheets). The first PARAFAC component is significantly negatively correlated with the activity of catalase, the ratio of proteins vs. phenols, and the number of ambrosia pollen particles (*p* < 0.05, Student’s *t*-test). PFSC2 is significantly positively correlated with the catalase activity, the protein-phenol ratio, and the ambrosia pollen content as well. A bit differently, PFSC1/PFSC2 is significantly correlated (*p* < 0.05, Student’s *t*-test) with the degree of infection and the number of the Caryophyllaceae pollen particles, both in a positive manner. PFSC2/PFSC1 is significantly correlated (*p* < 0.05, Student’s *t*-test) with the same variables but in a negative manner, which is expected. However, it is additionally significantly correlated with the number of Ligustrum pollen particles (*p* < 0.05, Student’s *t*-test). The degree of infection is statistically well correlated with the ratio of the PARAFAC scores, and with the amount of Ligustrum pollen. It is worth mentioning that the activity of diastase did not affect any of the measured parameters. The results indicate that catalase activity, the ratio of proteins vs. phenols, and the amount of Ambrosia pollen, Ligustrum pollen, and Caryophyllaceae pollen particles affect either the PFC1 or PFC2 spectral component.

### 4.5. PLS Modeling of the Spectral Features of Honey vs. Palynological and Physico-Chemical Properties

In order to pinpoint the individual contributions of each of the chemical, physico-chemical, and palynological properties to the spectral features of the tested samples, PLS modeling was performed. Four PLS models of different complexities were built for the first two spectral PARAFAC variables and their ratios. The models’ statistical performance parameters and complexities are summarized in Table 3. All of the models demonstrate good statistical performance and satisfactory predictive abilities at qualitative levels, with R^2^PRED in the range of 0.303 (*p* = 0.079, Student’s *t*-test) for modeling PFS2 to 0.654 (*p* < 0.05, Student’s *t*-test) for modeling the PFSC1/PFSC2 ratio. Although these values may be considered low for quantitative purposes, they are satisfactory for the qualitative interpretation of the models. The models are also similar in the number of used PLS components (mostly two, with one exception described by three PLS components). In most of them, the fifth honey sample exhibited a strong outlying effect, and in the case of PFSC2, the sample s10 did as well.

**Figure 4:**
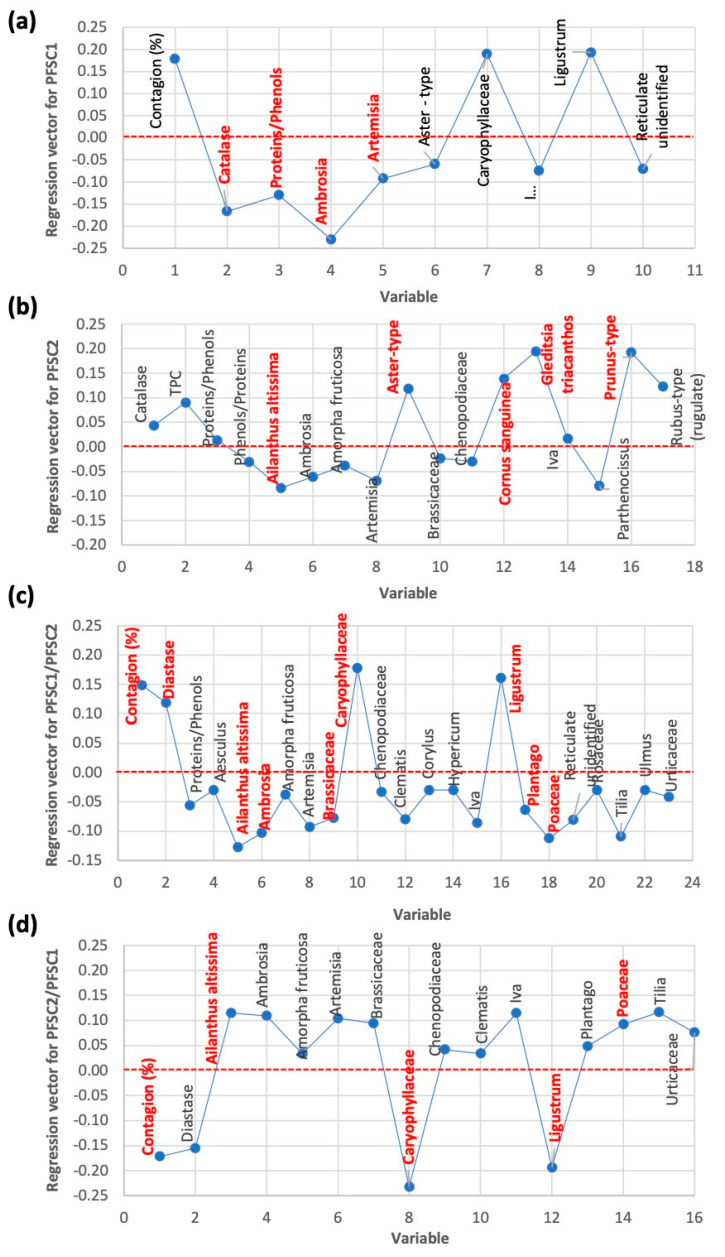
PLS regression vectors of models 1–4, where (**a**) PFSC1, (**b**) PFSC2, (**c**) PFSC1/PFSC2, and (**d**) PFSC2/PFSC1 are dependent variables. Variables that contribute to the final models better than average (VIP > 1) are marked in bold and red.

Regression vectors for all four dependent variables are depicted in Figure 4. It can be seen that PFSC1 was modeled by ten variables, out of which only catalase activity, protein/phenol ratio, and the number of ambrosia and artemisia pollen particles had VIP scores greater than 1, which means that they contribute to the model better than average. However, the regression coefficients associated with these variables are all negative. This is in accordance with our correlation analysis (Supplementary Table S1).

PFSC2 was successfully modeled by 17 variables, out of which only pollen particle contents of five species contribute to the model better than average: *Ailanths altissima*, *Aster-type*, *Cornus sanguinea*, *Gleditisia triacanthos*, and *Prunus* type. With the exception of *Ailanths altissima*, which has a negative regression coefficient, the others have a positive impact on PFSC2. These results may indicate that the pollen particles from the five mentioned species influence the PFC2 spectral component.

## 5. Conclusions

This study investigates the impact of *Nosema* infection on the physico-chemical and biochemical properties and spectral characteristics of honey, offering new insights into how colony health can affect honey quality. The results reveal that as *Nosema* infection levels increase in honeybee hives, significant changes occur in the water content, free acidity, optical rotation, and electrical conductivity of the honey, which reflect alterations in its biochemical composition. Specifically, honey from highly infected hives displayed higher water content, lower optical rotation, and reduced electrical conductivity, indicating potential shifts in sugar composition and the chemical environment of the honey.

Sugar analysis confirmed that honey from more infected hives contained lower glucose levels and higher sucrose concentrations, highlighting shifts in the sugar profile associated with infection. The principal component analysis (PCA) showed that honey from highly infected hives formed a distinct cluster in the score plot, primarily characterized by higher water content and lower optical rotation values, along with increased sugar content, particularly phenolic compounds.

PARAFAC modeling of fluorescence spectroscopy data provided further insights into the spectral contributions of proteins and phenolic compounds. The analysis revealed that *Nosema* infection levels significantly influence the relative abundances of these components, with higher infection levels correlating with increased phenolic content and decreased protein levels. Correlation and PLS modeling further emphasized the relationships between spectral features and various physico-chemical and biochemical properties, such as catalase activity, protein-to-phenol ratios, and specific pollen contents.

These results underscore the importance of *Nosema* infection as a factor that can alter both the biochemical composition and spectral signature of honey. Certain measured parameters such as catalase activity, the ratio of proteins vs. phenols, and the number of pollen particles from particular plant species affect either PFC1 or PFC2 spectral components. These findings suggest that spectral analysis, combined with chemometrics, can serve as a powerful tool for assessing honey quality and correlating honey quality parameters with the varying infection levels of the corresponding hives. Future research could further refine these models by leveraging fluorescence spectroscopy as a rapid and sensitive method while expanding the dataset to enhance predictive accuracy. This approach offers valuable tools for monitoring honey quality and bee health.

The study underscores the potential of fluorescence spectroscopy paired with chemometric methods as a groundbreaking, non-invasive approach for evaluating honey quality and detecting Nosema infections. These insights are invaluable for improving bee health monitoring practices and advancing sustainability in the beekeeping and honey production industries.

## Figures and Tables

**Table 1:** Results of the physico-chemical parameters of honey samples from hives with varying degrees of *Nosema* infection (negative optical rotation denotes left-handed; positive denotes right-handed honey). The values are shown as the mean ± SE.

Honey Sample	Hive Infection Level (%)	Water Content (%)	Free Acidity (meq/kg)	Specific Optical Rotation (°)	Electrical Conductivity at 20 °C (mS/cm)
1	0.00	13.10 ± 0.64	31.83 ± 1.74	−30.25 ± 1.71	0.60 ± 0.04
2	10.00	14.70 ± 0.82	24.08 ± 1.03	−25.75 ± 1.11	0.54 ± 0.03
3	13.33	14.85 ± 0.86	25.76 ± 1.22	−24.25 ± 1.34	0.57 ± 0.04
4	23.33	15.00 ± 0.69	20.64 ± 1.07	−5.25 ± 0.03	0.43 ± 0.03
5	30.00	16.20 ± 0.94	13.76 ± 0.74	1.50 ± 0.08	0.22 ± 0.02
6	43.33	16.30 ± 0.99	13.61 ± 0.67	−1.50 ± 0.09	0.22 ± 0.02
7	63.33	16.35 ± 0.92	12.72 ± 0.54	1.25 ± 0.04	0.22 ± 0.02
8	100.00	17.30 ± 1.04	13.85 ± 0.72	−1.25 ± 0.06	0.23 ± 0.02

**Table 2:** The average value of sugar content in honey samples (g/kg) from infected hives. The values are shown as the mean ± SE.

Honey Sample	1	2	3	4	5	6	7	8
Hive Infection Level (%)	0.00	10.00	13.33	23.33	30.00	43.33	63.33	100.00
Glucose	343.6 ± 17.3	301.8 ± 16.1	313.7 ± 15.9	324.7 ± 16.4	311.9 ± 16.2	246.5 ± 12.3	244.0 ± 12.1	278.3 ± 14.2
Fructose	404.2 ± 20.8	318.8 ± 16.3	311.6 ± 16.1	326.3 ± 16.9	427.9 ± 22.2	390.2 ± 20.3	395.2 ± 20.1	427.9 ± 23.1
Sucrose	28.9 ± 1.5	33.9 ± 1.8	53.6 ± 2.7	40.5 ± 2.1	42.8 ± 2.2	36.1 ± 1.9	37.1 ± 1.9	54.3 ± 2.8
Trehalose	0.44 ± 0.02	0.32 ± 0.02	0.14 ± 0.01	1.08 ± 0.06	0.97 ± 0.05	0.14 ± 0.01	0.64 ± 0.03	0.48 ± 0.03
Melibiose	0.19 ± 0.01	0.44 ± 0.02	0.04 ± 0.01	0.33 ± 0.02	0.24 ± 0.01	0.67 ± 0.04	0.21 ± 0.01	0.64 ± 0.03
Isomaltose	30.11 ± 1.51	9.52 ± 0.49	68.31 ± 3.51	7.62 ± 0.42	9.44 ± 0.49	1.63 ± 0.08	3.98 ± 0.22	19.13 ± 0.99
Turanose	6.21 ± 0.34	15.54 ± 0.79	3.04 ± 0.17	9.77 ± 0.46	7.13 ± 0.34	10.14 ± 0.53	8.58 ± 0.44	8.22 ± 0.44
Maltose	14.88 ± 0.81	12.24 ± 0.64	7.73 ± 0.42	8.62 ± 0.44	13.23 ± 0.72	2.52 ± 0.16	3.97 ± 0.22	15.92 ± 0.81
Melesitose	2.48 ± 0.15	0.33 ± 0.17	3.34 ± 0.19	4.09 ± 0.24	5.19 ± 0.28	4.36 ± 0.24	5.14 ± 0.28	5.08 ± 0.26

**Table 3:** Parameters of statistical performance of the PLS models. nPLS—number of PLS components used to build the model. NVar—number of variables finally included in the model after selection.

Model	Dependent	Statistical Parameters of Model Performance	Outliers
No.	Variable	*R*2Cal, *R*2CV, *R*2Pred, *RMSE*Cal, *RMSE*CV, *RMSE*Pred, nPLS, and NumbVar	
1	PFSC1	0.870, 0.705, 0.614, 119162, 192235, 208639, nPLS = 2, NVar = 10	
2	PFSC2	0.951, 0.806, 0.303, 30089, 71783, 565992, nPLS = 2, NVar = 17	S05, S10
3	PFSC1/PFSC2	0.952, 0.575, 0.654, 0.0276, 0.0933, 0.0774, nPLS = 2, NVar = 23	S05
4	PFSC2/PFSC1	0.955, 0.409, 0.367, 0.0259, 0.1286, 0.1093, nPLS = 3, NVar = 16	S05

## Data Availability

The original contributions presented in the study are included in the article/Supplementary Material; further inquiries can be directed to the corresponding author.

## Supplementary Materials

The following supporting information can be downloaded at: https://www.mdpi.com/article/10.3390/foods14040598/s1. Table S1: Correlation coefficient between infection with N. ceranae and analyzed enzymes, proteins, phenols, PARAFAC components, and concentrations of different pollen types that dominate in the honey samples.

## Abbreviations

The following abbreviations are used in this manuscript:

PCA	Principal Component Analysis
PARAFAC	Parallel Factor Analysis
PLS	Partial Least Squares regression
SE	Standard Error
