# Supplementary material for: Investigating the Impact of Nosema Infection in Beehives on Honey Quality Using Fluorescence Spectroscopy and Chemometrics
Source: Foods. 2025 Feb 11;14(4):598. doi: 10.3390/foods14040598 (PMC11853889; doi:10.3390/foods14040598)
Supplement: Supplementary file 1 [file foods-14-00598-s001.zip › foods-3428176-supplementary.pdf]

Table S1. Correlation coefficient between infection with *N. ceranae*, analyzed enzymes, proteins, phenols, PARAFAC components, and concentrations of different pollen types which dominate in honey samples.

	Infection (%)	*Catalase*	*Diastase*	*Proteins*	*TPC*	*Proteins/Phenols*	*Phenols/Proteins*	*PFSC1*	*PFSC2*	*PFSC1/PFSC2*	*PFSC2/PFSC1*	*Acer*	*Aesculus*	*Ailanthus altissima*	*Ambrosia*	*Amorpha fruticosa*	*Apiaceae*	*Artemisia*	*Aster-tip*	*Brassicaceae*	*Carduus tip*
Infection (%)	1,0	0,1	0,1	-0,4	-0,2	0,1	-0,2	0,4	-0,1	0,7	-0,7	-0,1	-0,1	-0,5	-0,1	-0,2	-0,1	0,0	-0,1	-0,2	-0,1
Catalase	0,1	1,0	-0,2	0,2	0,6	0,9	-0,9	-0,7	0,7	-0,3	0,2	0,5	0,4	0,0	0,8	-0,1	0,5	0,6	0,3	0,0	0,5
Diastase	0,1	-0,2	1,0	0,2	0,3	-0,6	0,2	0,4	-0,2	0,4	-0,4	0,1	0,2	0,1	-0,3	0,3	0,0	-0,7	-0,5	0,3	0,1
Proteins	-0,4	0,2	0,2	1,0	0,8	0,2	-0,4	-0,2	0,2	-0,1	0,2	-0,1	0,1	-0,1	0,1	0,2	0,0	0,1	0,4	0,1	0,0
TPC	-0,2	0,6	0,3	0,8	1,0	0,4	-0,6	-0,3	0,4	-0,1	0,1	0,1	0,3	0,0	0,3	0,3	0,1	0,3	0,4	0,3	0,0
Proteins/Phenols	0,1	0,9	-0,6	0,2	0,4	1,0	-0,9	-0,7	0,7	-0,4	0,3	0,3	0,2	-0,1	0,8	-0,2	0,3	0,9	0,6	-0,1	0,2
Phenols/Proteins	-0,2	-0,9	0,2	-0,4	-0,6	-0,9	1,0	0,5	-0,5	0,1	-0,1	-0,3	-0,3	0,2	-0,6	0,1	-0,4	-0,6	-0,3	0,0	-0,3
PFSC1	0,4	-0,7	0,4	-0,2	-0,3	-0,7	0,5	1,0	-0,8	0,7	-0,7	-0,5	-0,2	-0,2	-0,8	0,3	-0,5	-0,7	-0,5	0,1	-0,5
PFSC2	-0,1	0,7	-0,2	0,2	0,4	0,7	-0,5	-0,8	1,0	-0,1	0,2	0,6	0,0	-0,2	0,7	-0,6	0,5	0,6	0,6	-0,5	0,6
PFSC1/PFSC2	0,7	-0,3	0,4	-0,1	-0,1	-0,4	0,1	0,7	-0,1	1,0	-1,0	-0,2	-0,4	-0,6	-0,4	-0,3	-0,1	-0,4	-0,1	-0,5	-0,1
PFSC2/PFSC1	-0,7	0,2	-0,4	0,2	0,1	0,3	-0,1	-0,7	0,2	-1,0	1,0	0,2	0,3	0,6	0,4	0,3	0,2	0,4	0,1	0,5	0,1

	Caryophyllaceae	*Castanea sativa*	*Chenopodiaceae*	*Clematis*	*Cornus sanguinea*	*Corylus*	*Fabaceae*	*Fraxinus ornus*	*Gleditsia triacanthos*	*Hedera helix*	*Helianthus annuus*	*Humulus*	*Hypericum*	*Iva*	*Lamiaceae hexazonocolpate*	*Lamiaceae trizonocolpate*	*Ligustrum*	*Liliaceae*
Infection (%)	0,8	0,2	-0,1	-0,4	-0,2	-0,1	0,3	0,3	-0,4	-0,1	0,3	-0,1	0,0	0,1	-0,1	-0,1	0,8	-0,1
Catalase	-0,1	0,1	0,6	-0,2	-0,2	0,5	-0,4	-0,4	0,1	-0,1	-0,1	-0,1	0,3	0,6	0,5	0,5	0,0	0,5
Diastase	0,4	0,0	0,1	0,3	0,3	0,2	0,0	-0,3	0,5	-0,5	-0,3	-0,5	0,1	-0,7	0,1	0,1	0,4	0,1
Proteins	-0,4	-0,3	0,0	0,2	0,6	0,1	-0,2	-0,2	0,5	0,2	-0,3	0,2	0,1	0,0	-0,1	0,0	-0,3	-0,1
TPC	0,0	-0,4	0,1	0,3	0,4	0,3	-0,4	-0,6	0,4	-0,1	-0,5	-0,1	0,3	0,3	0,1	0,1	0,0	0,1
Proteins/Phenols	-0,2	0,0	0,3	-0,3	-0,2	0,3	-0,3	-0,2	-0,1	0,1	0,1	0,1	0,1	0,8	0,3	0,3	-0,1	0,3
Phenols/Proteins	0,0	0,0	-0,4	0,3	0,2	-0,4	0,3	0,2	0,0	-0,2	0,0	-0,2	-0,2	-0,5	-0,3	-0,4	-0,1	-0,3
PFSC1	0,6	-0,2	-0,5	0,1	0,0	-0,3	0,2	0,4	-0,3	0,3	0,0	0,3	0,0	-0,6	-0,5	-0,5	0,6	-0,5
PFSC2	-0,2	0,2	0,5	-0,3	0,3	0,1	-0,3	-0,4	0,6	-0,3	0,0	-0,3	-0,2	0,5	0,6	0,6	-0,4	0,6
PFSC1/PFSC2	0,8	0,0	-0,2	-0,4	0,3	-0,4	0,0	0,1	0,1	0,1	0,0	0,1	-0,3	-0,4	-0,2	-0,1	0,6	-0,2
PFSC2/PFSC1	-0,8	0,1	0,2	0,3	-0,2	0,4	0,1	-0,1	0,0	0,0	0,0	0,0	0,3	0,3	0,2	0,2	-0,7	0,2

	Parthenocissus	*Plantago*	*Poaceae*	*Potentila tip*	*Prunus-tip*	*Ranunculaceae*	*Reticulate unidentified*	*Rosaceae*	*Rubus-tip (rugulate)*	*rugulate unidentified*	*Salix*	*Saxifragaceae*	*Taraxacum-tip*	*Taxaceae/Cupressaceae*	*Tilia*	*Trizonocolporate reticulate*	*Ulmus*	*Unidetified_1*	*Unidentified_2*	*Urticaceae*	*Vitis*	*Zea*
Infection (%)	0,1	-0,3	-0,3	-0,2	-0,3	-0,1	0,1	0,0	0,0	-0,1	-0,3	-0,1	-0,3	-0,1	-0,4	-0,1	0,0	-0,2	-0,2	-0,1	-0,1	-0,2
Catalase	-0,3	0,4	0,6	0,3	-0,1	0,5	0,5	0,3	0,4	0,5	0,3	0,5	-0,2	0,5	-0,5	-0,1	0,3	-0,2	-0,1	0,6	0,5	-0,2
Diastase	0,1	0,4	0,1	-0,3	0,2	-0,1	-0,7	0,1	-0,5	0,1	0,3	0,1	0,3	0,1	-0,2	-0,5	0,1	-0,1	-0,4	0,2	0,1	0,3
Proteins	-0,1	0,3	0,1	0,2	0,6	0,1	0,0	0,1	0,1	-0,1	0,3	-0,1	0,6	-0,1	-0,5	0,2	0,1	0,6	0,3	0,0	-0,1	0,6
TPC	-0,2	0,5	0,4	0,0	0,4	0,0	0,2	0,3	0,1	0,1	0,4	0,1	0,4	0,1	-0,5	-0,1	0,3	0,3	-0,1	0,2	0,1	0,4
Proteins/Phenols	-0,3	0,1	0,4	0,2	-0,1	0,3	0,8	0,1	0,6	0,3	0,1	0,3	-0,2	0,3	-0,3	0,1	0,1	-0,1	0,0	0,3	0,3	-0,2
Phenols/Proteins	0,2	-0,3	-0,4	-0,4	0,0	-0,4	-0,5	-0,2	-0,4	-0,3	-0,2	-0,3	0,2	-0,3	0,7	-0,2	-0,2	0,0	-0,2	-0,4	-0,3	0,2
PFSC1	0,2	-0,3	-0,6	-0,1	-0,1	-0,4	-0,6	0,0	-0,5	-0,5	-0,3	-0,5	0,0	-0,5	0,0	0,3	0,0	0,2	0,3	-0,5	-0,5	0,0
PFSC2	-0,5	0,1	0,4	0,1	0,4	0,4	0,5	-0,2	0,5	0,6	0,3	0,6	0,2	0,6	-0,2	-0,3	-0,2	-0,1	-0,2	0,4	0,6	0,3
PFSC1/PFSC2	-0,3	-0,5	-0,5	0,0	0,3	-0,1	-0,3	-0,3	-0,1	-0,2	-0,3	-0,2	0,1	-0,2	-0,4	0,1	-0,3	0,2	0,1	-0,4	-0,2	0,3
PFSC2/PFSC1	0,3	0,5	0,5	0,1	-0,2	0,1	0,3	0,3	0,1	0,2	0,3	0,2	-0,1	0,2	0,3	0,0	0,3	-0,1	-0,1	0,3	0,2	-0,2

The values of Pearson's correlation coefficient greater than 0.632 and lower than -0.632 were considered to imply statistically significant correlation (marked in red) between variables at the predefined significance of p = 0.05. PFC1, PFC2–PARAFAC components.
